# The Effects of Intensive Residential Treatment for Feeding and Eating Disorders (FEDs) in Adolescence: The Case of an Italian Facility

**DOI:** 10.3390/nu17111904

**Published:** 2025-06-01

**Authors:** Valentina Lorenzoni, Francesca Casti, Gianluca D’Arcangelo, Linda Balluchi, Fabrizio Minichilli, Olivia Curzio, Sandra Maestro

**Affiliations:** 1 Institute of Management of the Scuola Superiore Sant’Anna, 56127 Pisa, Italy; valentina.lorenzoni@santannapisa.it; 2 Child and Adolescent Rehabilitation Clinic “Orti di Ada”, 56128 Pisa, Italy; francesca.casti1410@gmail.com (F.C.); gianluca.darcangelo@alice.it (G.D.); lindaballuchi@gmail.com (L.B.); sandra.maestro@hotmail.com (S.M.); 3 “San Giovanni Bosco” Advanced School of Educational Sciences (SED), 50121 Florence, Italy; 4 Institute of Clinical Physiology, National Research Council, 56124 Pisa, Italy; fabrizio.minichilli@cnr.it

**Keywords:** feeding and eating disorders (FEDs), adolescence, intensive residential treatment, evaluation study, longitudinal study, recovery

## Abstract

Background: Feeding and eating disorders (FEDs) represent a global health problem with an increasing incidence and a progressively earlier onset. Residential treatment is notable for its intensity and ability to provide multidisciplinary support to both patients and families. The objective of this study was to clinically characterize patients and to evaluate the impact of treatment at the “Orti di Ada” facility on the evolution of FEDs in adolescent patients. Methods: A cohort of 47 minors, treated in 2019–2024, was studied through longitudinal observation. Data were collected from medical records, and standardized questionnaires were administered at baseline (T0) and at the conclusion of treatment (T1). Comparisons between scores on the scales were made using paired t-tests for within-group changes from T0 to T1 or using Mann–Whitney tests for between-group comparisons. Spearman’s correlation coefficient was used to assess the relationship between pairs of variables. Results: The sample consisted of female patients (mean age: 15 years). The mean body mass index (BMI) at T1 was 16.6 kg/m^2^. The majority of patients (74.5%) had been undergoing treatment for less than one year. Most patients had been diagnosed with restrictive-type anorexia nervosa (74.5%), while 53.2% had multiple concomitant psychiatric comorbidities. The mean BMI increased to 18.7 kg/m^2^, suggesting biological recovery, along with the restoration of the menstrual cycle. Conclusions: Psychological measures showed significant improvements in subjects with exclusive depressive comorbidity. The correlations of age and disease duration with changes in questionnaire scores suggest that earlier treatment leads to more favorable outcomes. The results provided insights into the appropriateness of intensive treatment that, when targeting specific psychological factors, improves biological and psychological recovery.

## 1. Introduction

Feeding and eating disorders (FEDs) represent one of the most pervasive health issues between adolescents and young people in Western countries [1,2]. It is estimated that more than three million people suffer from these diseases in Italy and tens of millions of young people and adults are affected by FEDs all over the world every year [3]. Moreover, the COVID-19 pandemic has further exacerbated the situation, whereby new cases have increased between 30% and 35%, the age of onset was lowered, and there was an increase in new cases in males. These disorders typically arise during adolescence, and they frequently persist in adulthood. Their clinical complexity and the importance of an integrated, multidisciplinary treatment approach is related to the frequent co-occurrence of other psychiatric conditions, i.e., mood disorders—particularly major depressive disorder—and anxiety disorders [4]. In addition, eating disorders are often associated with a wide range of somatic conditions, affecting nearly every organ system (particularly the gastrointestinal and the reproductive systems). This broad spectrum of physical complications further emphasizes the systemic nature of eating disorders and the critical importance of ongoing medical monitoring and multidisciplinary care [5,6,7]. FEDs lead to serious outcomes, sometimes to the point of compromising the survival of the individual, due to their significant impact on physical health and on psychosocial well-being. The rapid increase in new cases has had major consequences both for the health of those affected and for available resources. Health services have been faced with large social and health care costs [8]; therefore, it is important to act as soon as possible to limit the consequences of these disorders. If properly treated, FEDs can go into remission within 3–4 years with appropriate treatment, leading to stable recovery in 70–80% of cases, with or without residual mild symptoms. The remission rate of anorexia nervosa (AN) varies between 20% and 30% within 2–4 years after onset, and between 70% and 80% after 8 or more years. The remission rate of bulimia nervosa (BN) is about 27% within one year of onset, and is over 70 percent after 10 or more years [9].

The current standard of care for anorexia nervosa (AN) is grounded in evidence-based interventions that prioritize medical stabilization through nutritional rehabilitation [10]. In pediatric populations, family-based treatment (FBT) has emerged as the most widely endorsed approach [11]. Despite structured therapeutic modalities, response rates vary significantly and relapse remains common. It is estimated that 20–30% of individuals go on to develop a severe and enduring form of the disorder, characterized by persistent symptoms and marked functional impairment [12]. Mortality rates associated with AN are among the highest of any psychiatric condition, with significantly elevated risk being observed in both female and male populations. Common causes of death include the direct physiological consequences of prolonged malnutrition—such as cardiac arrhythmias or multi-organ failure—as well as suicide, reflecting the substantial psychological distress often experienced by affected individuals [13,14]. This therapeutic stagnation can be partially attributed to a limited understanding of the complex and interwoven factors that contribute to the etiology and maintenance of anorexia nervosa. The interplay between genetic predisposition, neurobiological alterations, environmental stressors, and sociocultural influences presents a multifaceted challenge for researchers and clinicians [15]. Bridging these gaps in knowledge through interdisciplinary research is essential for the development of more effective interventions and for mitigating the considerable public health burden posed by this life-threatening disorder [16,17].

In 2017, the National Institute for Health and Care Excellence (NICE) released a revised comprehensive update of their guidelines regarding the identification, assessment, monitoring, and treatment of FEDs in children (0–12 years), adolescents (13–17 years), and adults. In Italy, the treatment for FEDs involves the following five different levels of intervention [18]: 1. general practitioner or freely chosen pediatrician; 2. outpatient treatment; 3. intensive or semi-residential outpatient treatment; 4. intensive residential rehabilitation; and 5. ordinary and emergency hospitalizations. These different levels of treatment make up a complex care network, which needs to be structured both longitudinally and transversally. Moreover, it is necessary that protocols for treatment, referral, and resumption of care are validated and shared by all providers involved in the treatment pathway [8]. It is recommended that treatment starts with the lowest level of care and only progresses to a more intensive option if no improvement is seen, allowing for a gradual and targeted approach. According to the Mental Health Information System (SISM) report of 2022, the term “residential facility” refers to a non-hospital setting where therapeutic and rehabilitation programs are applied and where people with psychiatric problems, referred by mental health centers, are treated according to a personalized plan that is regularly monitored [19]. FED rehabilitation becomes essential when the gravity of the disease is severe and associated with comorbidities, as well as when the quality of the patient’s life is compromised.

Residential treatment facilities for individuals with eating disorders have emerged as an increasingly prominent component of the continuum of care, offering an alternative to inpatient hospitalization. These settings are often positioned as providing intensive, recovery-oriented care within a less restrictive and more home-like environment, designed to support both psychological healing and nutritional rehabilitation. Unlike acute hospital settings, which tend to focus on medical stabilization and symptom containment, residential programs aim to foster longer-term recovery through integrated, multidisciplinary approaches that address the complex interplay of the physical, emotional, and psychosocial dimensions of eating disorders. In this context, recovery is not solely the result of clinical interventions aimed at eliminating disordered behaviors but is also the outcome of therapeutic environments that prioritize empowerment, relational support, and individualized care pathways. Thus, the implementation of person-centered and recovery-oriented principles within residential treatment settings may play a pivotal role in facilitating sustainable and meaningful recovery. These approaches not only respect the lived experience of the individual but also align with broader public health goals of promoting long-term mental health and social re-integration. Incorporating such models into residential care holds promise for enhancing treatment adherence, improving quality-of-life outcomes, and supporting the development of personal resilience. As public health systems increasingly recognize the burden of eating disorders and the limitations of traditional treatment modalities, there is a pressing need to evaluate and optimize residential care frameworks through rigorous outcome research and participatory models of service design [20]. The intensive rehabilitation program should possess certain key elements, including assessing the patient’s actual condition, as well as identifying overall targets and specific targets. The objectives change and develop according to the stage of the pathway in order to manage medical and psychiatric complications that may arise during treatment. Lastly, in order to collect data at different levels of intervention, it is necessary to obtain evidence supporting the development of guidelines orienting the practitioners in the decisional tree, leading to an integrated and complete approach to patient care. The present research was carried out at the “Orti di Ada” (“Ada’s vegetable gardens”), a high-intensity therapeutic–rehabilitation residential facility for minors that suffer from FEDs in Tuscany, Italy; they also carry out therapeutic projects aided by horticultural therapy [21]. The hypothesis underlying the present work is that residential treatment may produce significant benefits both with respect to medical stabilization—through nutritional rehabilitation—and in improving psychiatric comorbidities [22]. Residential treatment takes on specific significance as it addresses psychological dimensions such as interpersonal insecurity, low self-esteem, and fear of maturity that represent risk and maintenance factors for the eating disorder [23]. Community living provides a context in which patients can experience authentic relationships and mutual support. Such an environment fosters socialization, a sense of belonging, and the development of interpersonal skills and emotional regulation. Participation in shared routines and group dynamics can help young patients gradually cope with the fear of growth and the loss of control [24]. The clinical evolution of adolescents with anorexia nervosa is a very complex process that could evolve in many different ways. The course of the disorder can range from full recovery to chronic illness, with high variability in treatment response. Adequate intervention plays a critical role in improving outcomes. The aim of this observational prospective study was to evaluate the clinical evolution of an inception cohort of adolescents affected with FEDs treated in an intensive residential program. To our knowledge, this is the first study evaluating the outcomes of an intensive residential treatment in adolescents with FEDs. In particular, dimensions such interpersonal insecurity, self-esteem, and fear of maturity, as well as specific vulnerabilities of FEDs in adolescents, may benefit from the community life experienced during residential intervention.

## 2. Materials and Methods

### 2.1. Setting

“Orti di Ada”, in central Italy, is a high-intensity therapeutic–rehabilitation facility for minors affected with feeding and eating disorders (FEDs), who are referred mainly in the post-acute phase. The patients’ age for admission to the program is between 8 and 17 years old. The facility offers daily clinical monitoring; meal support and post-meal supervision; individual, family, group psychotherapy; psycho-educational groups; and other expressive activities, which aim to restore the healthy physical and psychological condition of patients and to provide support to families. The facility also provides daily school activities that allow the adolescent to maintain contact with aspects of social life typical of their stage of development [18].

### 2.2. Study Design

A longitudinal observational research design was chosen to evaluate the evolution of adolescents affected by FEDs treated in an intensive residential program. A common set of measurements was collected at the time of patient admission (T0), as well as at their discharge (T1), to be able to evaluate changes over time and to assess the impact of the treatment. This study was conducted in accordance with the principles outlined in the Declaration of Helsinki [25] and was approved on April 16th 2019 by the Regional Ethical Committee of Meyer Hospital (Florence, Italy), number 64/2019. Each parent or legal tutor of the minor subjects provided written informed consent before their enrollment.

### 2.3. Measures

Demographics and biological measures: Specific demographic and biological measures were collected at enrollment (age, BMI, and status of menstrual cycle) and at discharge (duration of treatment, dietary adherence, BMI, and status of menstrual cycle). These measures are crucial in evaluating the clinical evolution of FEDs as adolescents are in a critical stage of physical and emotional development, which can influence both the onset of the disorder and its treatment outcomes. In detail, while age affects growth potential and the body’s response to malnutrition and recovery, BMI is a direct indicator of nutritional status and body weight. BMI is often used to assess the degree of underweight and monitor weight restoration during treatment. A significant change in BMI during treatment can be an important marker for progress or deterioration in health. Moreover, BMI helps evaluate the risk of medical complications related to malnutrition.

The status of the menstrual cycle is a critical biological indicator in female adolescents and adults with anorexia nervosa. A loss of menstruation (amenorrhea) is a common consequence of malnutrition and weight loss, and its presence or absence can reflect the severity of the condition. The restoration of menstrual function often indicates that the body is recovering from the physiological stress caused by the eating disorder, and it is closely linked to improvements in nutritional status and hormonal balance.

Disease and treatment history: The duration of the eating disorder plays a significant role in the prognosis of anorexia nervosa. Chronicity can lead to more entrenched patterns of disordered eating and greater medical complications, making recovery more difficult. A long duration of illness is also associated with higher risks of developing severe and enduring anorexia nervosa (SE-ED), which may be more resistant to treatment.

Comorbid psychiatric disorders, such as depression, anxiety, obsessive–compulsive disorder, and substance abuse, are common in patients with anorexia nervosa. The presence of comorbidities complicates the clinical course and treatment of AN. For example, individuals with depression or anxiety may have a harder time engaging in therapeutic interventions, leading to poorer outcomes. Understanding the nature and severity of these comorbidities is essential in assessing their clinical evolution and providing a holistic treatment plan.

The duration of treatment is an important measure of clinical progress. Long treatment durations may be necessary for patients with more severe forms of anorexia nervosa, while shorter treatment durations may indicate a quicker recovery or less-severe cases. However, extended treatment periods may also be indicative of more entrenched symptoms, relapse, or difficulty adhering to dietary changes and therapeutic interventions.

Dietary adherence is a key factor in the treatment of anorexia nervosa, as nutritional rehabilitation is fundamental to recovery. Failure to adhere to prescribed dietary plans can lead to continued malnutrition and hinder weight restoration. Monitoring dietary adherence provides insight into how well patients are engaging with the treatment and whether psychological or behavioral issues (such as resistance to food intake) are interfering with recovery.

Psychological measures: The EDI-3, CAPS, and BUT questionnaires were used to assess the psychological profiles of adolescents.

The Eating Disorder Inventory-3 (EDI-3) questionnaire is a self-assessment tool that provides clear profiles of individuals’ functioning. Its purpose is to monitor the psychological variables and symptoms involved in the development and maintenance of the disorder. The EDI-3 questionnaire has been validated across clinical and non-clinical populations, showing that it effectively measures the psychological constructs associated with eating disorders. Scores on the EDI-3 subscales correlate well with diagnostic categories and symptom severity in individuals with eating disorders. Cronbach’s alpha for most subscales is >0.80, indicating good consistency of items within each scale. The EDI-3 showed stable results over time when conditions do not change significantly. The EDI-3’s multidimensional structure helps track changes in core psychopathological traits during treatment [26]. All EDI-3 subscales were obtained and the scores were converted to T-scores using the conversion table provided in the Garner and Giannetti manual. Composite scores were then calculated and entered into the study database in a systematic manner [26].

The Child and Adolescent Perfectionism Scale (CAPS) aims to assess the two main dimensions of perfectionism in young people—self-oriented perfectionism (SOP) and socially prescribed perfectionism (SPP). The Child and Adolescent Perfectionism Scale (CAPS) measures perfectionism in youth, specifically distinguishing between self-oriented (internal standards) and socially prescribed (external pressure) perfectionism. The content validity is strong—items directly reflect theoretical components of perfectionism. Confirmatory factor analyses support a two-factor model (SOP and SPP), validating the CAPS structure. The internal consistency is good—Cronbach’s alpha for subscales typically ranges between 0.74 and 0.86. The test–retest reliability ranged from being moderate to high in adolescent samples. Using separate subscale scores (SOP, SPP, and total) enhances the precision in identifying how perfectionism operates in relation to eating disorder symptoms. Perfectionism is a known maintenance factor in anorexia nervosa, making this tool especially relevant [27]. SOP, SPP, and the total score, which adds both together, were considered. This procedure allowed for a clear view of the different dimensions of perfectionism [27].

The Body Uneasiness Test (BUT) assesses body image dissatisfaction, preoccupations, and discomfort, which are key factors in the psychopathology of eating disorders. This test makes it possible to assess dissatisfaction with specific parts of the body or bodily functions, as well as any general feeling of discomfort that would be difficult to describe in verbal terms. The content validity is strong—the items comprehensively cover physical and psychological aspects of body dissatisfaction. The construct validity was supported by factor analyses. The BUT has shown good sensitivity in distinguishing between clinical and non-clinical populations. The internal consistency is high—Cronbach’s alpha for the GSI and subscales is usually above 0.85. The test–retest reliability is strong—repeated measures within short intervals are consistent. The Global Severity Index (GSI) is an excellent overall indicator of body-related distress, which can be tracked across treatment phases. The GSI score was calculated by adding the values of the 34 responses and by calculating the arithmetic mean. This provided an overall indication of the severity of the symptoms reported by the patients The BUT helps in detecting subtle changes in body image perception that might not be easily verbalized by adolescents [28].

### 2.4. Study Sample

All female patients taking part in intensive residential treatment at the “Orti di Ada” between 2019 and 2024 were considered for inclusion in this study; there were also two male subjects among the patients, but they were not included in the research analysis. A total of 47 subjects were included in the study; for some of them, the full set of psychological measures was not available at both admission and discharge (N = 13). The analysis of the evolution of patients in terms of biological condition was thus performed considering the whole sample of subjects recruited; an additional analysis was also performed focusing on the sub-sample with a complete psychological assessment at both T0 and T1 to assess the evolution of the psychological profile.

### 2.5. Statistical Analysis

Quantitative measures (such as age) were described using mean and standard deviation (SD) in the case of variables with symmetric distribution or using median and range of variability (minimum and maximum values) in the case of non-symmetric variables or in the case of the description of subgroups of limited size. Categorical or dichotomous variables were described by reporting the number of subjects, and the corresponding relative frequency was expressed as a percentage. A comparison of the scores for the scales of the different questionnaires used in the study at T0 and T1 was performed using the Student T-test for paired data or the Wilcoxon test, as appropriate. Comparisons of the subgroups of subjects were performed considering the independent Student T-test or the Mann–Whitney test, depending on the distribution of variables that was evaluated using the Kolmogorov–Smirnov test. Spearman’s correlation coefficient was used to evaluate the relationship between pairs of variables; the correlation between pairs of variables was reported graphically by representing the statistically significant correlations in the matrix graph and highlighting the positive correlations in red and the negative ones in blue. The significance level was set to 0.05 and all analyses were performed with R software version 4.5.0.

## 3. Results

### 3.1. Characterization of the Full Sample

A total of 47 patients were admitted to and treated in the Child and Adolescent Rehabilitation Clinic “Orti di Ada” between 2019 and 2024. All patients were female with an average age at the time of admission of 15 (std. dev. = 1.7) years. The average BMI at admission was 16.6 (std. dev. = 2.7) kg/m^2^, indicating underweight and, hence, a condition of moderate severity. Less than half of the patients (42.6%) were diagnosed with an FED between 1 and 2 years before enrollment, 29.8% were diagnosed 3 or more years before entry, and the remaining 25.5% were diagnosed less than a year before. The majority of the sample (74.5%) suffered from restrictive anorexia nervosa, 17% presented with the binge/purging variant, while 4.3% was diagnosed with bulimia. As regards comorbidities, 85.1% of patients presented with associated comorbidities. In particular, the associated disorders detected were depressive disorder, anxiety disorder, and personality disorders that were spread out as follows: 53.2% of patients presented with multiple concomitant disorders; 27.7% suffered from depression; 8.5% did not show any comorbidities; and 4.3% showed personality disorders. Furthermore, 74.5% of participants received treatment for less than a year, 21.3% received treatment for between 1 and 2 years, and only 2.1% received treatment lasting longer than 2 years (Table 1).

### 3.2. Clinical Evolution of the Full Sample

The average BMI significantly increased (*p*-value < 0.001) at discharge, rising to 18.7 (std. dev. = 2.2) kg/m^2^, corresponding to a normal weight and, thus, to a remission of the clinical condition. Therefore, all the patients in the sample with secondary amenorrhea recovered their menstrual cycle except three (6.4%) pre-pubertal subjects that, as a condition of primary amenorrhea, suffered from menarche during the period spent in the residential facility.

### 3.3. Characterization of the Sub-Sample

Among all the patients admitted to the clinic, 34 (72.3%) accomplished baseline and follow-up assessment, completing all the scales foreseen for the evaluation of the psychological profile. That subgroup was considered for the analysis of the evolution of the psychological and psychopathological traits of the disease.

As is shown in Table 1, the characteristics of the subgroup did not differ from those of the overall patients presenting to the clinic. The sub-sample is made up of females, with an average age of approximately 15 (std. dev. = 1.6) years at the time of entry and whose clinical evolution showed a significant increase (*p*-value < 0.001) in BMI (as compared to enrollment), with average values at discharge being 18.7 (std. dev. = 1.9) kg/m^2^ (Table 1).

### 3.4. Evolution of the Psychological Profile in the Sub-Sample

**Figure 1:**
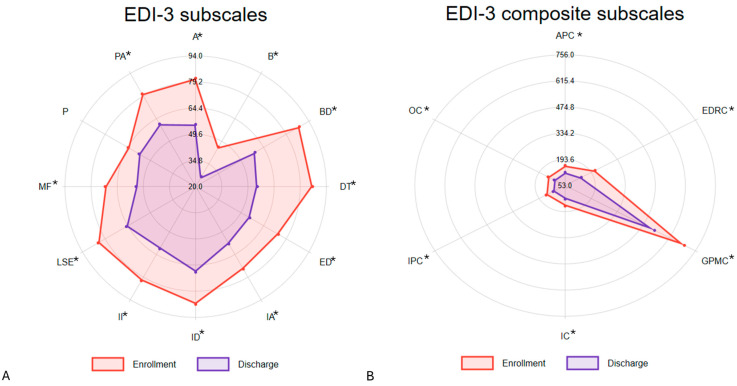
Mean EDI-3 scales at the time of admission and discharge. * *p*-value < 0.05. The figure shows the mean values of the EDI-3 subscales (panel **A**) and composite subscales (panel **B**) at the time of admission and at discharge (after treatment). All values decreased significantly (except the perfectionism (P) subscale), suggesting an improvement in psychological condition.

The mean values of the EDI-3 scales at both enrollment and discharge in the sub-sample with complete psychological measures are shown in Figure 1.

Table 2 details all the scores obtained from the instruments used at enrollment and after treatment completion in the sub-sample. All scales significantly decrease (with a *p*-value less than 0.05) after treatment completion, except for the perfectionism scale (P) of the EDI-3 and the CAPS questionnaire. A significant decrease, suggesting an improvement in psychological condition, was observed for all the EDI-3 subscales, except for perfectionism and for BUT. No statistically significant change was observed for any of the CAPS subscales.

To understand the possible impact of comorbidities on the evolution of the psychological measures, we explored the evolution in the group of patients with only comorbid depression (N = 11), comparing it with those with multiple comorbidities (N = 21). The two groups showed no differences in the change in BMI (average difference from enrollment being 2.5 [2; 4.1] among those with depression and 2.1 [1.5; 3.7] among those with multiple comorbidities; *p*-value = 0.427). Moreover, subjects with comorbid depression showed a greater improvement over time in comparison with those with multiple comorbidities, specifically in the following EDI-3 scales: body dissatisfaction (BD), eating disorder risk composite (EDRC), low self-esteem (LSE), interpersonal insecurity (II), interoceptive deficit (ID), ineffectiveness composite (IC), affective problems composite (APC), and global psychological maladjustment (GPMC). For CAPS scores, the differences between T0 and T1 were not statistically significant (Table 3).

When comparing patients with a treatment duration of less than 1 year (N = 26) with those treated for a longer period (N = 8), no significant differences were found both in clinical evolution (BMI differences being 2.6 [2; 3.8] and 1.5 [0.7; 3.2], respectively; *p*-value = 0.350) and in the evolution of the psychological profile, except for the following EDI-3 scales: emotional dysregulation (ED) and maturity fear (MF). The first decreased to a greater extent in those patients undergoing a longer treatment, while the latter decreased in those with a treatment duration of less than a year and increased in those with a longer treatment duration (Table 4).

### 3.5. Correlations Between Clinical and Demographic Characteristics at Enrollment and the Evolution of Psychological Measures

**Figure 2:**
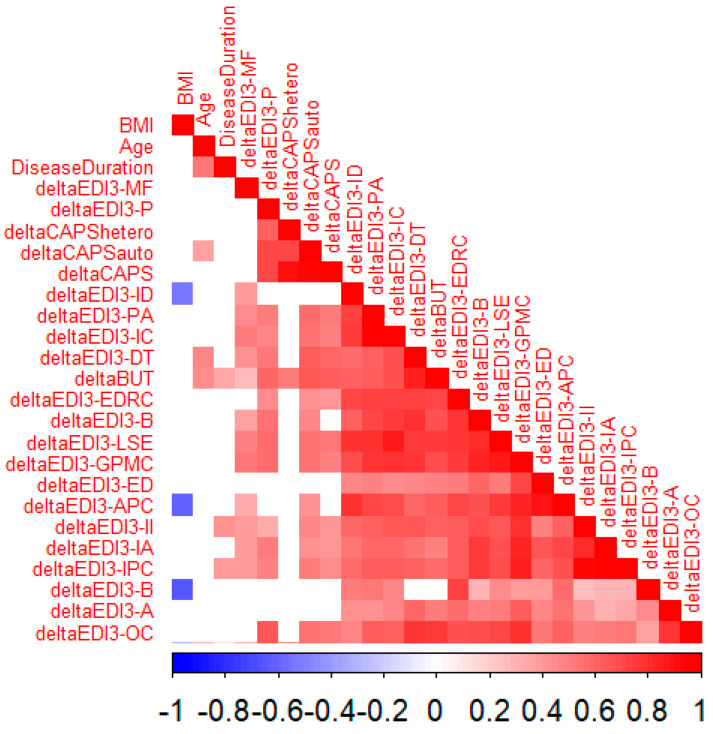
Pairwise correlation between the main characteristics at enrollment and changes in psychological measures at discharge (from enrollment).

As is shown in Figure 2, a significant negative correlation was found between the entry BMI and changes in some measures, e.g., the score on the bulimia (B) (rho = −0.641; *p*-value = 0.009), affective problems composite (APC) (rho = −0.602; *p*-value = 0.049), and interoceptive deficit (ID) (rho = −0.525; *p*-value = 0.022) scales was lower for higher subject entry BMI values. No significant correlations emerged between the entry BMI and the other scales assessed. There was also a significantly positive correlation between patients’ entry age and some of the changes in psychological measures, suggesting that the higher the age of entry, the lower the improvement in the CAPS auto (rho = 0.364; *p*-value = 0.041) in the BUT questionnaire (rho = 0.460; *p*-value = 0.047), as well as in the drive for thinness (DT) scale (rho = 0.460; *p*-value = 0.026) in the EDI-3 questionnaire. Instead, no significant correlations emerged between the age of entry and the other scales assessed. Finally, a longer disease duration was associated with higher scores on the interpersonal insecurity (II) (rho = 0.421; *p*-value = 0.028) and interpersonal problems composite (IPC) (rho = 0.240; *p*-value = 0.033) scales of the EDI-3, as well as with the BUT questionnaire (rho = 0.350; *p*-value = 0.044).

The figure shows statistically significant correlations; blue cells suggest a significantly negative correlation between variables, while a red color was used to highlight positive correlation. The intensity of the color is proportional to the strength of the correlation.

## 4. Discussion

The aim of this study was to explore the characteristics and clinical progression of adolescents with FEDs undergoing high-intensity residential treatment. The data from the sample align with both national and international guidelines for the residential treatment of FEDs. These patients, although not in acute clinical conditions, display high levels of ego-syntonic thinking related to the disorder, which is compounded by psychiatric comorbidities. Previous treatments of a lower intensity had not produced the desired results and the risk to the patients’ health was increasing, involving physical, psychiatric, or psychosocial risks. The sample primarily consisted of individuals in early adolescence [29,30], with restrictive anorexia nervosa being the predominant diagnosis (74.5%), as well as a high prevalence of depressive and anxiety comorbidities.

Biological conditions and physiological dimensions

Although biological conditions were not extremely severe, factors like amenorrhea or delayed pubertal development due to neuroendocrine mechanisms hindered brain maturation, interrupting the hormonal influence that is crucial for adolescent brain growth. Key factors such as amenorrhea and delayed pubertal development, often resulting from neuroendocrine dysregulation, impede the hormonal processes that are essential for typical brain growth and connectivity. The hormonal insufficiency contributes to delayed or absent puberty [31,32]. Adolescents with AN can exhibit alterations in brain structure and function, particularly in areas associated with the “social brain,” such as the medial prefrontal cortex and ventral striatum. Even after weight restoration, some neurocognitive deficits persist, suggesting that the developmental window for certain brain functions may have been compromised. Understanding the interplay between neuroendocrine function and brain maturation is essential for developing comprehensive treatment strategies [33].

It is noteworthy that by the end of treatment, all patients in the present study had regained their menstrual cycle, and three pre-pubertal subjects had their first menstruation. This improvement can be partly attributed to the biological recovery observed at the end of treatment (average BMI: 18.7), as well as to the effects of communal living, which promote peer-to-peer comparisons and social skill development while influencing the endocrine balance.

Treasure et al. suggested that the earlier the disorder emerges, the more severe the consequences, if early intervention is not provided. Emerging evidence underscores the idea that the earlier the onset of anorexia nervosa, the more severe the potential consequences, particularly if early intervention is not provided [34]. This assertion is supported by longitudinal studies indicating that delayed treatment initiation is associated with poorer long-term outcomes. A study assessing the long-term outcomes of adolescent-onset AN found that a significant duration of untreated illness before admission was a strong predictor of current eating disorders, with an odds ratio of 3.334 (*p* = 0.014). This highlights the critical importance of early detection and intervention in improving prognosis [35]. Furthermore, research indicates that an early response to psychological treatment is a robust predictor of post-treatment outcomes in AN [36]. The severity of anorexia nervosa is significantly influenced by the age of onset and the timeliness of intervention. The present research data confirmed the benefit of earlier intervention, in particular for subjects with an early-onset disorder; moreover, even if we have found a subgroup that may require longer treatment, our data suggest that a one-year treatment duration may be effective for the majority of patients. Despite being limited to the study sample, these findings may offer insight into the organization of FED treatment in other contexts, as it seems that even with a relatively short duration of treatment, in residential settings, it is possible to achieve favorable outcomes.

Psychological and psychopathological dimensions

The present study was able to assess changes in the psychological and psychopathological dimensions of FEDs through self-reports (EDI-3, BUT, and CAPS) at the beginning and end of treatment in 73% of the sample. The results showed significant improvement in nearly all explored dimensions, supporting the positive clinical and psychological evolution of patients undergoing residential treatment. Specifically, the following EDI-3 scales improved: body dissatisfaction (BD), eating disorder risk composite (EDRC), low self-esteem (LSE), interpersonal insecurity (II), interoceptive deficit (ID), ineffectiveness composite (IC), affective problems composite (APC), and global psychological maladjustment composite (GPMC). These data align with the existing literature, which identifies body dissatisfaction as a key symptom of anorexia nervosa and bulimia nervosa [37].

An exception to this improvement was the perfectionism scale on the EDI-3, which was confirmed by scores on the CAPS, particularly regarding self-reported perfectionism. Many studies identify perfectionism as a personality trait that predisposes individuals to develop anorexia nervosa, especially the restrictive type, and contributes to its persistence. In the trans diagnostic model, clinical perfectionism is seen as a maintaining mechanism influencing eating, weight, and body image control, which further strengthens self-esteem and the sense of effectiveness [38]. Perfectionism is also labeled as a maintaining factor in the study of Curzio et al., who explore the applicability of the trans diagnostic model in a sample of 417 children and adolescents [32].

Standard interventions could be insufficient to address this complex trait; perfectionism is a significant risk factor for the onset and maintenance of eating disorders, and its persistence during treatment can impede recovery. Recent studies have highlighted the need for targeted interventions to address perfectionism in this population. Perfectionism encompasses both “strivings” (the pursuit of high standards) and “concerns” (fear of making mistakes and negative evaluation). To effectively address perfectionism in adolescents with FEDs, integrating specialized interventions into residential treatment may be beneficial. Enhanced Cognitive–Behavioral Therapy (CBT-E) is an evidence-based treatment for eating disorders that focuses on the underlying psychological processes contributing to the disorder. It has been found to be effective and consists of structured sessions, often including homework and active client participation. Incorporating components that specifically target perfectionism within CBT-E may enhance treatment outcomes [39,40]. Schema therapy addresses deep-seated patterns or themes that develop during childhood and are elaborated throughout an individual’s life. These schemas can influence behavior and emotional regulation. A feasibility trial developed a parent-supported CBT for perfectionism in adolescents with eating disorders, which was found to be acceptable and feasible, with no attrition reported. Additionally, a systematic review and meta-analysis indicated that perfectionism interventions were effective in reducing perfectionism and disordered eating, with large effect sizes [41]. These findings underscore the importance of integrating targeted interventions that address perfectionism into residential treatment programs for adolescents with FEDs.

Our study also found that psychological improvement was significantly correlated with the age of the patients and illness duration. Specifically, patients who started treatment at a younger age and with a shorter illness duration showed greater improvement in psychological and psychopathological dimensions. This is consistent with existing studies, which suggest that the earlier the intervention, the more likely recovery is to be successful [34]. Early intervention prevents certain personality traits from transforming from predisposing to maintaining factors, such as perfectionism, interpersonal insecurity, and fear of maturity. Regarding intervention timeliness, data from the Maudsley family therapy trial suggest that delayed interventions, especially for those with a long illness duration, yield poorer results [42].

Disease history and treatment duration

Regarding disease duration, the present analysis showed that most patients had suffered from FEDs for one to two years (44.1%), a significant proportion (38.2%) had been diagnosed for more than two years, and 17.7% had been diagnosed less than a year ago. Those with a disease duration of over two years tend to have a worse outcome. The correlation between disease duration and self-report scores showed that a longer illness duration is associated with higher levels of interpersonal insecurity and problems, reflected in the EDI-3 and BUT scales. These findings suggest that as the disease becomes more chronic, psychological aspects like insecurity and interpersonal difficulties worsen, complicating recovery. Our data are aligned with evidence suggesting that a longer illness duration before treatment and a low BMI are linked to poorer outcomes, underscoring the importance of early diagnosis and intervention [43,44].

Finally, another interesting fact concerns the duration of treatment. In this study, no statistically significant changes were observed between subjects who completed treatment within one year and those whose treatment program lasted longer than 12 months. However, for older subjects, treatment lasting more than a year was associated with significantly improved outcomes on two important dimensions—body dissatisfaction and maturity fears. This seems to indicate that more time is needed to influence these two very typical features of eating disorders. Concerning psychiatric comorbidities, our results showed that patients with only depressive disorder as a comorbidity improved more significantly compared to those with multiple psychiatric comorbidities. Notably, patients with multiple comorbidities, such as depression and anxiety, showed less improvement.

Study limitations

Some limitations should be acknowledged for the present study. First of all, the present study is an exploratory investigation related to patients treated in the center of interest. No sample size calculation has been performed and results cannot be easily generalized to other setting as the sample considered cannot be considered representative of FED patients attending residential treatment. The results obtained are important to design future multicenter studies. In this regard, the research group [21,45] is pursuing other clinical and health management studies to increasingly optimize and broaden study methods and results. Other limitations of the present study are related to the lack of a control group and of a longitudinal follow-up after discharge, which is needed to assess sustained recovery. These limitations should be considered in future studies.

## 5. Conclusions

Research on the evolution of eating disorders (EDs) in patients undergoing residential treatment remains limited. This study evaluates changes in ED patients and provides an initial overview of their progress. However, further research is necessary to better understand the evolution of patients in residential care and to identify the most effective treatment strategies. The study also compares the outcomes of residential treatment with inpatient and outpatient approaches, which typically involve family participation.

The outcome of anorexia nervosa is influenced by several factors, including body mass index (BMI), physical risk, age, and illness duration. Recovery becomes less likely the longer the illness persists, highlighting the importance of early intervention, particularly before weight loss becomes too severe or prolonged. A landmark study from the Maudsley family in the 1980s demonstrated that patients who returned to a normal weight after initial inpatient treatment and who received early family therapy showed significantly greater weight gain at the one-year follow-up. At the five-year follow-up, the results indicated that family therapy had lasting positive effects. However, if the illness duration exceeded three years, family therapy was no more effective than individual therapy, with both being linked to poor outcomes. This underscores the importance of early intervention with effective treatments like family therapy [42].

In conclusion, the present study aimed to monitor changes in patients between T0 and T1 using self-assessment questionnaires (EDI-3, BUT, and CAPS) administered upon admission and discharge. The study initially involved 47 female patients, primarily diagnosed with restrictive anorexia nervosa, and with high rates of comorbidities, particularly depressive and anxiety disorders. Most patients had been ill for one to two years, while the remaining patients had been affected for over two years, suggesting a medium-to-high clinical severity. The study found that a longer illness duration was associated with poorer outcomes, particularly regarding insecurity and interpersonal issues, which is likely due to the social isolation that tends to intensify as the disorder becomes chronic. Despite these challenges, the results were largely positive, with significant improvements in both physical (BMI increased by 12.7%) and psychological aspects. However, several issues remain that require further investigation, such as treatment duration, as no significant differences were observed between patients treated for less than a year and those treated for longer periods. Additionally, the role of perfectionism, which is a core symptom of anorexia nervosa, warrants further exploration. Despite various limitations, the results from our study serve as a basis for future research; moreover, although our findings cannot be easily generalized to other settings, they may serve as an input to organize and plan the management and treatment of FEDs in other contexts. In particular, our findings suggest the need to improve the ability to provide early detection and treat patients, as well as suggesting that enhancing the availability of residential treatment, even offering relatively short treatment, could be crucial to restoring the biological and psychological and social conditions of adolescents with FEDs.

## Figures and Tables

**Table 1:** Baseline characteristics of the study samples *.

	Full Sample (N = 47)	Sub-Sample with Full Psychological Assessment (N = 34)
Age, mean (std. dev.)	15 (1.7)	15 (1.6)
Gender	47 (100%)	34 (100%)
Living in Tuscany region		
No	23 (48.9%)	17 (50%)
Yes	22 (46.8%)	16 (47.1%)
Not known	2 (4.3%)	1 (2.9%)
BMI at enrollment	16.6 (2.7)	16.5 (2.9)
Disease duration		
<1 years	12 (25.5%)	6 (17.7%)
1–2 years	20 (42.6%)	15 (44.1%)
>3 years	14 (29.8%)	13 (38.2%)
Not known	1 (2.1%)	-
Diagnosis		
Restrictive anorexia	35 (74.5%)	26 (76.5%)
Binge/Purging	8 (17.0%)	7 (20.6%)
Bulimia	2 (4.3%)	1 (2.9%)
Not known	2 (4.3%)	-
Number of comorbidities		
None	4 (8.5%)	-
Depression	13 (27.7%)	11 (32.4%)
Anxiety	1 (2.1%)	1 (2.9%)
Personality disorder	2 (2.1%)	1 (2.9%)
More comorbidities	25 (53.2%)	21 (61.8%)
Not known	3 (6.4%)	-
Treatment duration		
<1 years	35 (74.5%)	26 (76.5%)
1–2 years	10 (21.3%)	7 (20.6%)
>3 years	1 (2.1%)	1 (2.9%)
Not known	1 (2.1%)	0

* Note: All data are presented in terms of number of subjects (percentage), except age, which was shown as mean (std. dev.).

**Table 2:** Psychological measures at enrollment (T0) and at discharge (T1).

	Enrollment (T0)	Discharge (T1)	*p*-Value
Drive for thinness (DT)	86.2 (24.3)	55.4 (37.3)	0.00005
Bulimia (B)	46.2 (29.7)	28.8 (29.9)	0.033
Body dissatisfaction (BD)	88.5 (14.8)	60.8 (35.7)	0.00016
Eating disorder risk composite (EDRC)	219.1 (57.4)	145 (90.5)	0.00012
Low self-esteem (LSE)	85 (19)	67.4 (31.4)	0.003
Personal alienation (PA)	81.6 (24.9)	63 (34.4)	0.020
Interpersonal insecurity (II)	82.7 (20.2)	61.2 (30.6)	0.00036
Interpersonal alienation (IA)	78.2 (27.8)	58.3 (30)	0.002
Interoceptive deficit (ID)	88.1 (15.7)	69.8 (25.1)	0.001
Emotional dysregulation (ED)	78 (21.7)	56.6 (28.8)	0.001
Perfectionism (P)	64.8 (27.3)	58.2 (34)	0.191
Ascetism (A)	82.8 (20.6)	55.9 (33.1)	0.00007
Maturity fear (MF)	69.4 (30.6)	53.7 (32.4)	0.028
Ineffectiveness composite (IC)	165.8 (38.3)	130.4 (64)	0.008
Interpersonal problems composite (IPC)	160.9 (45.7)	119.6 (57.4)	0.001
Affective problems composite (APC)	166.1 (33.1)	126.4 (47.3)	0.00011
Overcontrol composite (OC)	147.6 (42.4)	114.2 (53.7)	0.00026
Global psychological maladjustment (GPMC)	710.6 (138.2)	546.1 (222.5)	0.00031
BUT	3.4 (1)	2.1 (1.4)	0.00017
CAPS auto	42.6 (13.6)	41 (14.7)	0.490
CAPS hetero	29.5 (10.7)	27.6 (9.8)	0.459
CAPS total	72 (21)	68.7 (22.3)	0.435

**Table 3:** Changes in psychological measures at discharge (from enrollment) in those with depression compared to those with multiple comorbidities.

	Comorbid Depression (N = 11)	Multiple Comorbidities (N = 21)	*p*-Value
Drive for thinness (DT)	−58 [−85; −18]	−14 [−32; 0]	0.068
Bulimia (B)	−42 [−61; 0]	−27 [−57; 29]	0.450
Body dissatisfaction (BD)	−63 [−88; −10]	−6 [−22; 0]	0.025
Eating disorder risk composite (EDRC)	−184 [−213; −37]	−42 [−91; 28]	0.031
Low self-esteem (LSE)	−34 [−67; −16]	−3 [−11; 4]	0.015
Personal alienation (PA)	−68 [−89; 1]	−5 [−19; 11]	0.054
Interpersonal insecurity (II)	−34 [−69; −6]	−5 [−22; 0]	0.041
Interpersonal alienation (IA)	−41 [−64; 0]	−6 [−32; 2]	0.084
Interoceptive deficit (ID)	−32 [−60; −11]	−5 [−23; 0]	0.007
Emotional dysregulation (ED)	−46 [−64; −2]	−16 [−29; 3]	0.057
Perfectionism (P)	−8 [−45; 3]	−6 [−18; 7]	0.475
Ascetism (A)	−28 [−79; −12]	−15 [−36; 0]	0.197
Maturity fear (MF)	−23 [−71; −8]	−5 [−33; 1]	0.153
Ineffectiveness composite (IC)	−95 [−153; 1]	−4 [−25; 14]	0.045
Interpersonal problems composite (IPC)	−68 [−138; −6]	−9 [−48; 6]	0.054
Affective problems composite (APC)	−66 [−119; −22]	−21 [−55; −4]	0.022
Overcontrol composite (OC)	−43 [−76; −31]	−12 [−38; 2]	0.059
Global psychological maladjustment (GPMC)	−422 [−495; −63]	−94 [−188; 32]	0.018
BUT	−1.6 [−3.6; −0.2]	−0.9 [−1.9; 0.2]	0.302
CAPS auto	−5 [−17; 11]	0 [−15; 8]	0.642
CAPS hetero	−4.5 [−12; 10]	1 [−12; 7]	1.000
CAPS total	−11 [−29; 21]	0 [−31; 11]	0.816

**Table 4:** Changes in psychological measures at discharge (from enrollment) in those remaining on treatment for less than one year as compared to those staying longer.

	Lenght of Treatment < 1 Year (N = 26)	Lenght of Treatment >= 1 Year (N = 8)	*p*-Value
Drive for thinness (DT)	−22 [−72; −1]	−9 [−20.5; −1]	0.329
Bulimia (B)	−16 [−52; 21]	−40.5 [−65; 0]	0.339
Body dissatisfaction (BD)	−14 [−63; 0]	−5.5 [−23.5; 0]	0.477
Eating disorder risk composite (EDRC)	−56 [−184; 19]	−70 [−84.5; −39]	0.984
Low self-esteem (LSE)	−8.5 [−51; 2]	−3 [−11.5; 9.5]	0.149
Personal alienation (PA)	−13 [−68; 11]	−1 [−16; 25.5]	0.273
Interpersonal insecurity (II)	−15.5 [−60; −3]	−1 [−9; 3]	0.074
Interpersonal alienation (IA)	−13 [−46; 0]	−5 [−41; 8.5]	0.405
Interoceptive deficit (ID)	−15.5 [−33; −3]	−2.5 [−19; 10]	0.207
Emotional dysregulation (ED)	−15 [−38; 11]	−43.5 [−57.5; −18.5]	0.049
Perfectionism (P)	−6 [−25; 6]	2 [−32.5; 22.5]	0.583
Ascetism (A)	−17 [−43; 0]	−23 [−48.5; −6.5]	0.570
Maturity fear (MF)	−24.5 [−37; −5]	20.5 [−20; 55]	0.038
Ineffectiveness composite (IC)	−18 [−123; 13]	12.5 [−27.5; 35]	0.109
Interpersonal problems composite (IPC)	−28 [−106; −5]	−9 [−44.5; 13.5]	0.215
Affective problems composite (APC)	−25 [−66; 6]	−45.5 [−62; −16.5]	0.460
Overcontrol composite (OC)	−31.5 [−66; 2]	−34.5 [−66; −12.5]	0.903
Global psychological maladjustment (GPMC)	−112 [−401; 5]	−73 [−244.5; 40.5]	0.503
BUT	−14405 [−2683; 0.4]	−0.9 [−17215; 0.1]	0.730
CAPS auto	−4 [−15; 2]	6 [−7; 16]	0.200
CAPS hetero	−2 [−12; 4]	6 [−5; 11.5]	0.223
CAPS total	−7 [−31; 2]	15.5 [−9.5; 27]	0.165

## Data Availability

The data are not available due to privacy reasons, but they can be requested by accredited research institutions.
